# Supporting healthy ageing through community-based dietary education

**DOI:** 10.1016/j.jnha.2026.100932

**Published:** 2026-07-24

**Authors:** Annelie Turesson

**Affiliations:** Department of Food Studies, Nutrition and Dietetics, Uppsala University, Sweden

The number of older adults is increasing worldwide, making healthy ageing an important public health goal [1]. As people live longer, attention is shifting from simply extending lifespan to maintaining health, independence, and quality of life in later years [[1], [2], [3]]. Cardiometabolic diseases, including cardiovascular disease and type 2 diabetes, are among the most common health conditions affecting older adults and contribute substantially to disability, reduced quality of life, and healthcare burden [4]. Finding effective ways to prevent and manage these conditions is therefore essential for supporting healthy ageing.

Nutrition is increasingly recognised as a cornerstone of healthy ageing and cardiometabolic disease prevention [[4], [5], [6]]. There has been a shift from focusing on single nutrients to dietary patterns, as people do not consume isolated nutrients but rather combinations of foods and food groups [5,7]. Several dietary patterns rich in vegetables, fruits, whole grains, legumes, nuts, and unsaturated fats have consistently been associated with lower cardiometabolic risk and improved health outcomes across diverse populations [8]. Additionally, dietary patterns such as the Mediterranean diet [9,10], the Dietary Approaches to Stop Hypertension (DASH) diet [11], and the Nordic diet [12] have all been associated with reduced risk of cardiovascular disease.

However, translating nutritional evidence into sustainable dietary behaviour change among community-dwelling older adults remains challenging, as populations differ in their food cultures, eating habits, and broader social contexts [13,14]. Dietary behaviours are influenced by a complex interplay of individual, social, environmental, and cultural factors [15]. Therefore, dietary recommendations and interventions need to be adapted to local cultural contexts to enhance feasibility, relevance, and long-term adherence [8,13]. This highlights the important role of primary healthcare and community-based programmes in promoting and sustaining healthy dietary behaviours in everyday life [16]. However, despite growing recognition of the importance of cultural adaptation, evidence on the long-term effectiveness of such interventions across diverse populations remains limited [14].

This evidence gap is particularly important because community-based dietary interventions have shown promise for improving cardiometabolic health [17]. In this context, Wu et al. [18] demonstrated that a 12-month community-based nutrition education intervention promoting a cardiometabolic risk-reducing dietary pattern significantly improved cardiometabolic health among community-dwelling older adults in China. Compared with usual health education, participants in the intervention group experienced greater reductions in overall cardiometabolic risk, accompanied by improvements in blood pressure, lipid profile, glucose metabolism and body composition. The intervention was also associated with reduced progression of carotid atherosclerosis, suggesting potential long-term benefits for cardiovascular health. These findings support the feasibility and effectiveness of culturally adapted community-based dietary strategies for reducing cardiometabolic disease risk in older populations. Similar findings have been reported in other cardiovascular prevention programmes conducted in community settings [19].

One of the key messages from this body of research is that healthy eating should be viewed not only as an individual responsibility but also as a public health opportunity [6]. Community-based nutrition education may help translate research findings into practical and accessible dietary advice for older adults [17,19]. Importantly, programmes that incorporate local food traditions and eating habits appear to improve adherence to healthy dietary patterns and contribute to reductions in cardiometabolic risk among older adults [18,19].

Diets developed in one population, such as the Mediterranean diet, may not always be feasible or appropriate in other cultural settings [14]. Moreover, the American Heart Association emphasises that dietary recommendations should account for social, cultural, and environmental factors that influence food choices [6]. As populations continue to age, integrating culturally adapted dietary interventions into community and primary care settings may represent an important strategy for promoting healthy ageing and reducing cardiometabolic disease risk [8,18].

The findings support dietary education as a non-pharmacological strategy to improve cardiometabolic health and potentially contribute to healthy ageing and reduced healthcare burden [18]. A proposed conceptual framework linking dietary education, improved dietary behaviours, cardiometabolic health, and healthy ageing is illustrated in Fig. 1.

**Fig. 1:**
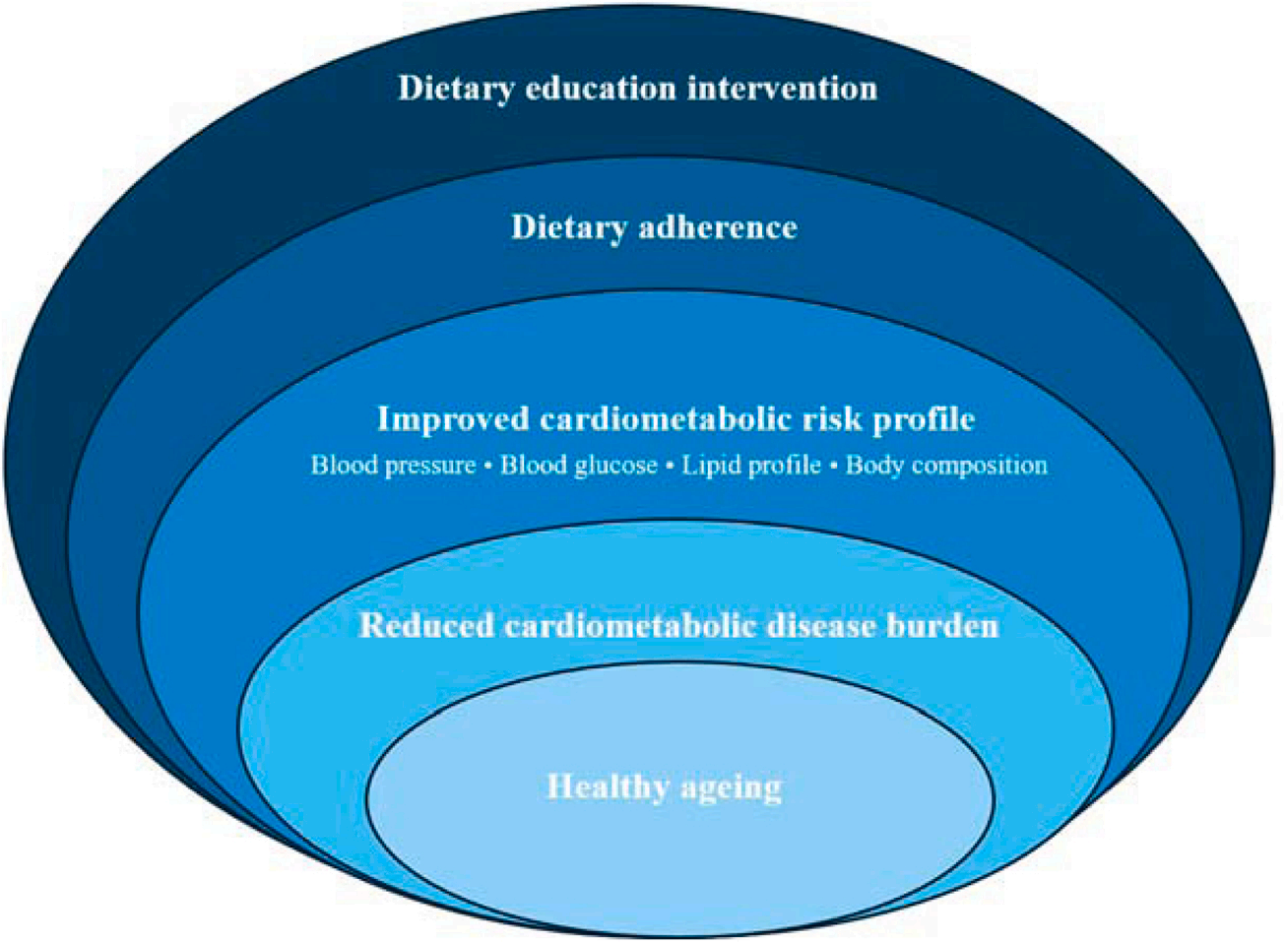
From dietary education to healthy ageing. Conceptual framework illustrating how dietary education interventions may promote dietary adherence, improve cardiometabolic risk profiles, reduce cardiometabolic disease burden, and support healthy ageing.

As ageing research increasingly shifts from disease management to the promotion of health and function, future studies should focus on sustainable and person-centred dietary strategies that help older adults maintain independence and everyday functioning [20]. Such approaches should recognize the diverse needs, preferences, and circumstances of older adults and support dietary changes that are both practical and meaningful. Beyond traditional cardiometabolic risk factors, future research should also consider outcomes such as physical function, cognitive health, well-being, and quality of life to capture a broader perspective on healthy ageing and the potential benefits of dietary interventions [21].

Taken together, these findings suggest that culturally adapted, community-based dietary interventions could play an important role in supporting healthy ageing and reducing the growing burden of cardiometabolic disease in older populations worldwide.

## Declaration of Generative AI and AI-assisted technologies in the writing process

During the preparation of this work, the author used Microsoft Copilot to assist with grammar and language refinement. The authors reviewed and edited all suggested changes and take full responsibility for the content of the published article.

## Funding

No funding was received for this work.

## Declaration of competing interest

The author declares no conflicts of interests.
